# Extract from the Records of the Atlanta Academy of Medicine, April 22, 1871

**Published:** 1871-05

**Authors:** W. A. Love

**Affiliations:** Corresponding Secretary


### *Extract from the Records of the Atlanta Academy of Medicine, April* 22, 1871.

Whereas it has been both the policy and the practice of the Atlanta Academy of Medicine as a body, and of its members individually, to conform strictly to the Code of Ethics of the American Medical Association in the effort to elevate our professional standard and to cultivate a knowledge of science, not desiring to interfere with others who may select different associations, or prefer different plans for the same objects; and,

Whereas, the subject of the association of others, or their operations in their individual or associate capacity, has never been entertained or alluded to within the Academy by any one of its members, either directly or indirectly; and,

Whereas, the Atlanta Academy of Medicine has been violently, and without notice, assailed before the Georgia Medical Association, at its late annual meeting in Americus, and many of its members misrepresented by certain individuals claiming to represent a Medical organization; and,

Whereas, the Academy having sent no delegate to that Association, and having no direct representation on the floor, when said assault was made on its members individually, seeking their professional, and therefore individual degradation; and,

Whereas, our President, Dr. J. P. Logan, did, as a member of the Association, expose the animus of the prosecutors, and defend the Academy against such unwarranted and unjustifiable attack; therefore,

*Resolved*, That our President, Dr. J. P. Logan, is justly entitled to the thanks of this Academy for such course, and the same is hereby tendered unanimously.

Unanimously adopted.

